# IFNγ Inhibits the Cytosolic Replication of *Shigella flexneri* via the Cytoplasmic RNA Sensor RIG-I

**DOI:** 10.1371/journal.ppat.1002809

**Published:** 2012-08-09

**Authors:** Stephanie P. Jehl, Catarina V. Nogueira, Xuqing Zhang, Michael N. Starnbach

**Affiliations:** Department of Microbiology and Immunobiology, Harvard Medical School, Boston, Massachusetts, United States of America; University of Michigan Medical School, United States of America

## Abstract

The activation of host cells by interferon gamma (IFNγ) is essential for inhibiting the intracellular replication of most microbial pathogens. Although significant advances have been made in identifying IFNγ-dependent host factors that suppress intracellular bacteria, little is known about how IFNγ enables cells to recognize, or restrict, the growth of pathogens that replicate in the host cytoplasm. The replication of the cytosolic bacterial pathogen *Shigella flexneri* is significantly inhibited in IFNγ-stimulated cells, however the specific mechanisms that mediate this inhibition have remained elusive. We found that *S. flexneri* efficiently invades IFNγ-activated mouse embryonic fibroblasts (MEFs) and escapes from the vacuole, suggesting that IFNγ acts by blocking *S. flexneri* replication in the cytosol. This restriction on cytosolic growth was dependent on interferon regulatory factor 1 (IRF1), an IFNγ-inducible transcription factor capable of inducing IFNγ-mediated cell-autonomous immunity. To identify host factors that restrict *S. flexneri* growth, we used whole genome microarrays to identify mammalian genes whose expression in *S. flexneri*-infected cells is controlled by IFNγ and IRF1. Among the genes we identified was the pattern recognition receptor (PRR) retanoic acid-inducible gene I (RIG-I), a cytoplasmic sensor of foreign RNA that had not been previously known to play a role in *S. flexneri* infection. We found that RIG-I and its downstream signaling adaptor mitochondrial antiviral signaling protein (MAVS)—but not cytosolic Nod-like receptors (NLRs)—are critically important for IFNγ-mediated *S. flexneri* growth restriction. The recently described RNA polymerase III pathway, which transcribes foreign cytosolic DNA into the RIG-I ligand 5′-triphosphate RNA, appeared to be involved in this restriction. The finding that RIG-I responds to *S. flexneri* infection during the IFNγ response extends the range of PRRs that are capable of recognizing this bacterium. Additionally, these findings expand our understanding of how IFNγ recognizes, and ultimately restricts, bacterial pathogens within host cells.

## Author Summary

*Shigella flexneri*, the major cause of bacillary dysentery worldwide, invades and replicates within the cytoplasm of intestinal epithelial cells, where it disseminates to neighboring cells and ultimately increases the likelihood of transmission to uninfected hosts. A hallmark of the mammalian immune system is its ability to inhibit the growth of such intracellular pathogens by upregulating intracellular resistance mechanisms in response to the cytokine IFNγ. We found that in non-myeloid host cells stimulated with IFNγ *S. flexneri* remains able to invade the cells efficiently and gain access to the host cytoplasm. Once in the cytoplasm of IFγ-activated cells, the RIG-I/ MAVS immunosurveillance pathway is activated, enabling the stimulated host cells to inhibit *S. flexneri* replication. Interestingly, RIG-I only played a minor role in the cellular response to this pathogen in the absence of IFNγ, suggesting that the IFNγ response ensures the recognition of the infection through an immunosurveillance pathway that is otherwise dispensable for controlling *S. flexneri* growth. Together, these findings implicate the RIG-I pathway as a crucial component in the cellular response to this devastating bacterial pathogen.

## Introduction

*Shigella flexneri* is a Gram-negative bacterial pathogen that causes bacillary dysentery, resulting in significant morbidity and mortality worldwide. Following ingestion, *S. flexneri* translocate through the colonic epithelial cell barrier, where they infect resident macrophages and rapidly induce caspase-1-dependent pyroptotic cell death in these cells [1], [2], [3]. After escaping from the dying macrophages, *S. flexneri* invade nearby colonic epithelial cells using a Type III secretion system (TTSS) and become temporarily enclosed within a membrane-bound vacuole. The bacteria rapidly escape from the vacuole using a poorly defined mechanism and enter the host cytoplasm, where they engage in both intra- and inter- cellular motility by inducing local actin polymerization at one pole of the bacterium [4]. Invasion, vacuole escape, and intercellular spreading augment the dissemination of *S. flexneri* throughout the epithelium. Simultaneously, however, these virulence mechanisms also inadvertently allow greater recognition of the bacterium by the host through various intracellular immunosurveillance pathways. The stimulation of these immunosurveillance pathways ultimately leads to the induction of a robust proinflammatory response and the eventual resolution of infection [5], [6], [7], [8].

A critical mediator of the proinflammatory response to *S. flexneri* is the cytokine IFNγ (also known as Type II IFN), which acts on a wide variety of cells types to regulate the expression of over 2,000 genes [9]. In the past decade, significant progress has been made in identifying and characterizing the downstream IFNγ-inducible intracellular resistance mechanisms that coordinate the killing or growth inhibition of microbial pathogens. Some of these mechanisms include the targeting of bactericidal reactive oxygen species (ROS) to pathogen containing vacuoles (PCVs), the direct vesiculation and destruction of PCVs [10], [11], [12], [13], and the induction of antimicrobial autophagy [14]. Although advances have been made in identifying IFNγ-inducible intracellular resistance mechanisms, the mechanisms responsible for restricting many cytosolic bacterial pathogens have largely remained elusive, presumably a result of redundancy among effector mechanisms. One study found that *Francisella tularensis* escapes to the cytosol of IFNγ-activated primary macrophages but is subsequently restricted for cytosolic growth by an unknown mechanism, independently of reactive nitrogen species (RNS) or ROS [15]. However, a parallel study found that inhibition of RNS was able to block *F. tularensis* killing but did not restore intracellular replication [16]. In contrast, the cytosolic pathogen *Listeria monocytogenes* fails to escape the phagosome and is subsequently killed in IFNγ-activated peritoneal macrophages due to the functional disruption of the hemolysin listeriolysin O (LLO) by RNS and ROS [17], [18].

The importance of IFNγ in host defense during *S. flexneri* infection was demonstrated by Way, et al., who showed that the lethal dose of *S. flexneri* is 5 logs greater in immunocompetent mice compared to IFNγ^−/−^ mice [9]. Furthermore, immunocompetent mice challenged with 10^5^ CFU of *S. flexneri* were able to clear the infection by 5 days post infection, while IFNγ^−/−^ mice were unable to inhibit *S. flexneri* replication and eventually succumbed to the infection. The effect of IFNγ on cell autonomous resistance to *S. flexneri* has also been demonstrated. Primary mouse macrophages or rat L2 fibroblasts pre-treated with IFNγ prior to infection significantly inhibit *S. flexneri* growth compared to untreated cells [9]. Although IFNγ is a critical mediator of innate immunity against this bacterium, the IFNγ-inducible host factors mediating cell autonomous resistance against this bacterium are completely unknown. Moreover, unlike *F. tularensis* and *L. monocytogenes*, no data are available on the specific step of *S. flexneri* pathogenesis that is blocked by IFNγ in macrophages or in other cell types also naturally infected by this bacterium, such as non-myeloid epithelial cells.

Here we sought to identify which step of *S. flexneri* intracellular infection is inhibited in IFNγ-activated non-myeloid cells and to begin to define the cellular mechanism(s) and pathways that are enabled by IFNγ to recognize or restrict intracellular *S. flexneri* infection. We found that *S. flexneri* efficiently invades and escapes from the vacuole of IFNγ-activated MEFs and are inhibited at the step of cytosolic replication. Furthermore, we found that the detection of *S. flexneri* infection by the cytoplasmic RNA sensor RIG-I was required for the inhibition of *S. flexneri* cytosolic growth by IFNγ. Interestingly, *S. flexneri* genomic DNA and RNA were sufficient to induce RIG-I dependent immune responses. Additionally, chemical inhibition of host RNA polymerase III partially blocked the ability of IFNγ to inhibit *S. flexneri* growth, suggesting that *S. flexneri* DNA is a stimulus of the IFNγ-dependent immune response against this bacterium. Collectively, these findings implicate the RIG-I/MAVS signaling pathway as a crucial component of cell autonomous IFNγ-mediated restriction of cytosolic bacterial pathogens.

## Results

### IFNγ restricts the cytosolic replication of *S. flexneri* in MEFs

**Figure 1:**
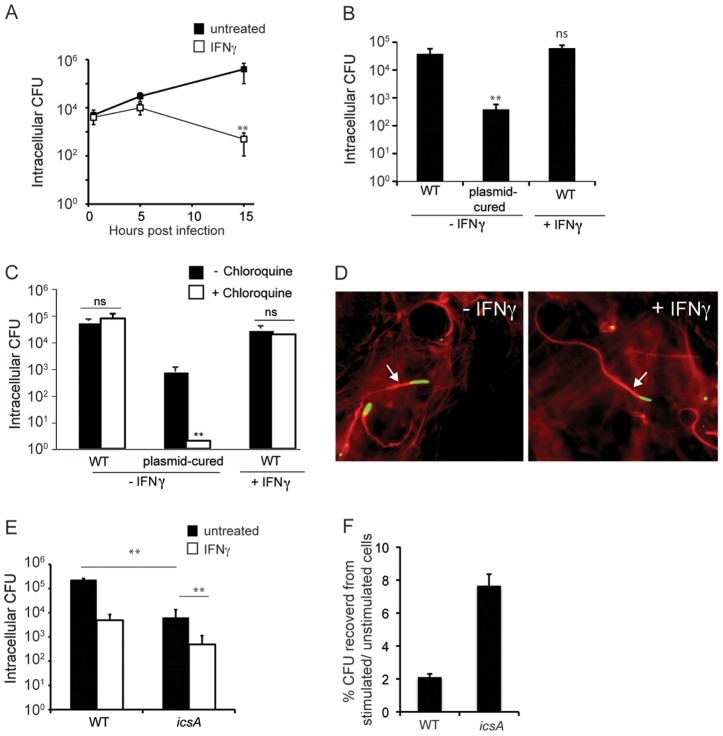
IFNγ inhibits the replication of *S. flexneri* in the host cytoplasm. (A) Growth of *S. flexneri* in MEFs that were unstimulated or stimulated with IFNγ for 24 hours prior to infection. (B) Quantification of *S. flexneri* invasion at 1 hpi. (C) Quantification of *S. flexneri* vacuole escape. Unstimulated or IFNγ-stimulated cells were infected and subsequently treated with gentamicin alone or gentamicin and choloroquine (50 µg/ml), as indicated. Bacterial CFU were determined at 2 hpi. (D) Fluorescence micrographs showing actin tail formation by *S. flexneri*. Unstimulated or IFNγ-stimulated MEFs were infected with GFP-expressing *S. flexneri* (green) and stained for host cell actin (red) at 2 hpi. White arrows indicate actin tails. Data for quantification of actin tail formation was gathered from at least 50 infected cells per condition, for 2 independent experiments. (E) Growth of WT or non-motile *icsA* in unstimulated or IFNγ-stimulated MEFs. MEFs were infected with WT or *icsA S. flexneri*, and CFU were determined at 15 hpi. (F) Normalization of CFU in IFNγ-stimulated cells for infections with different strains. CFU recovered from IFNγ-stimulated cells for each strain were normalized against CFU recovered from unstimulated cells. All data shown (A–C, E–F) are means and standard deviations. Experiments are representative experiments from 3 (A, B) or 2 (C–F) independent experiments. Where appropriate, significant statistical differences are indicated as follows: ns, not significant; *, p<0.05; **, p<0.005 (Student's t test). Unless indicated otherwise, noted statistical differences are between unstimulated and IFNγ-stimulated cells for each condition.

The replication of *S. flexneri* within the colonic epithelium is an essential determinant of this bacterium's pathogenesis. Previously it was demonstrated that IFNγ inhibits the growth of this bacterium in both mouse macrophages and rat L2 fibroblasts [9]. To determine how IFNγ might inhibit the intracellular growth of this bacterium in the epithelium, we examined *S. flexneri* growth in mouse primary MEFs, as a model for non-myeloid epithelial cells. While unstimulated MEFs were highly permissive for *S. flexneri* replication over a 15 hour infection, pre-stimulation of MEFs with IFNγ prior to infection drastically inhibited bacterial growth by 15 hours post infection (hpi) (Fig. 1A). Interestingly, *S. flexneri* grew similarly well in unstimulated and IFNγ-stimulated cells for at least 5 hpi, indicating that the antimicrobial mechanisms that block *S. flexneri* growth are not immediately felt by the bacteria. The addition of IFNγ to the cell culture media only at the time of the infection had no effect on *S. flexneri* growth (data not shown), suggesting that IFNγ-mediated priming of the cell prior to infection is necessary for the restriction.

Invasion of host cells and vacuolar escape by *S. flexneri* are essential for the evasion of extracellular and intracellular immune mechanisms, respectively. An intriguing hypothesis to explain the ability of IFNγ to inhibit intracellular *S. flexneri* is that this cytokine affects a specific stage of the bacterium's intracellular pathogenic cycle to prevent otherwise efficient escape from antimicrobial mechanisms. While a non-invasive virulence plasmid-cured strain of *S. flexneri* was over 100-fold less invasive than the wild-type (WT) strain, WT *S. flexneri* invaded unstimulated and IFNγ-stimulated cells with similar efficiencies (Fig. 1B). Many vacuolar– and even cytosolic– pathogens are killed in IFNγ-activated cells following invasion by mechanisms targeting nascent pathogen containing vacuoles formed during microbial invasion or uptake [18]. Therefore, one possibility was that IFNγ-induced mechanisms either destroy *S. flexneri* in the vacuole prior to vacuole escape or functionally disrupt the action of bacterial effectors necessary for vacuole escape. To assess the ability of *S. flexneri* to escape from the vacuole of IFNγ-activated MEFs, unstimulated and stimulated cells were infected for 30 minutes and subsequently treated with chloroquine, which concentrates in phagosomes of host cells at bactericidal levels. As expected, the plasmid-cured strain (which is deficient for vacuole escape) was killed in the presence of chloroquine, confirming that chloroquine effectively killed bacteria trapped in the vacuole. Interestingly, similar numbers of bacteria were recovered from unstimulated and stimulated MEFs both in the absence and presence of chloroquine, suggesting that *S. flexneri* efficiently escapes to the cytoplasm of IFNγ-treated cells (Fig. 1C). Collectively these findings demonstrate that *S. flexneri* enters into cells and accesses the cytoplasm of IFNγ-activated MEFs, demonstrating that inhibition occurs after the organisms reach the cytoplasm.

Once in the host cytoplasm, *S. flexneri* becomes motile and spreads to adjacent cells through the activity of IcsA, a bacterial cell surface-associated protein required for the polymerization of host actin in the cytoplasm [19], [20]. Since access to the cell cytoplasm is a prerequisite for actin tail formation, we examined the ability of *S. flexneri* to form actin tails at 2 hpi to confirm that *S. flexneri* reaches the cytoplasm of IFNγ-activated cells. *S. flexneri* formed actin tails in both untreated and IFNγ-treated cells with comparable frequency (30% and 26%, respectively) (Fig. 1D), confirming their presence within the cytoplasm. Although IFNγ did not inhibit the intracellular motility of *S. flexneri* at early time points, we hypothesized that a *S. flexneri* mutant that was unable to move might be more easily targeted by potential IFNγ-induced mechanisms and therefore be more susceptible to IFNγ-mediated killing. To test this hypothesis, we examined the survival of the non-motile *S. flexneri icsA* mutant, which is fully invasive but deficient for actin tail polymerization and motility [21]. We found that the *icsA* strain was more restricted for growth compared to the WT strain in unstimulated MEFs at 15 hpi, suggesting that non-motile *S. flexneri* are more efficiently targeted by IFNγ-independent mechanisms (Fig. 1E). Additionally, like the WT strain, Δ*icsA* was significantly inhibited by IFNγ stimulation. However, when these data were normalized against the observed IFNγ-independent killing (CFU recovered from IFNγ-stimulated cells/CFU recovered from unstimulated cells), *icsA* was not more susceptible to IFNγ-mediated killing compared to the WT strain (8% *icsA* versus 2% WT recovered from IFNγ-treated cells over untreated cells). This suggests that the mechanisms that inhibit *S. flexneri* replication during the IFNγ response are not specifically targeted to non-motile bacteria. The finding that *icsA* is significantly inhibited by IFNγ also indicates that inhibition of *S. flexneri* occurs intracellularly, by a mechanism that does not depend on the escape or spreading of *S. flexneri* to the extracellular space or to other cells. Collectively, these experiments demonstrate that, in non-myeloid cells, *S. flexneri* growth is inhibited by IFNγ following bacterial invasion and vacuolar escape, at the stage of cytosolic replication.

### Restriction of *S. flexneri* growth by IFNγ is dependent on IRF1

**Figure 2:**
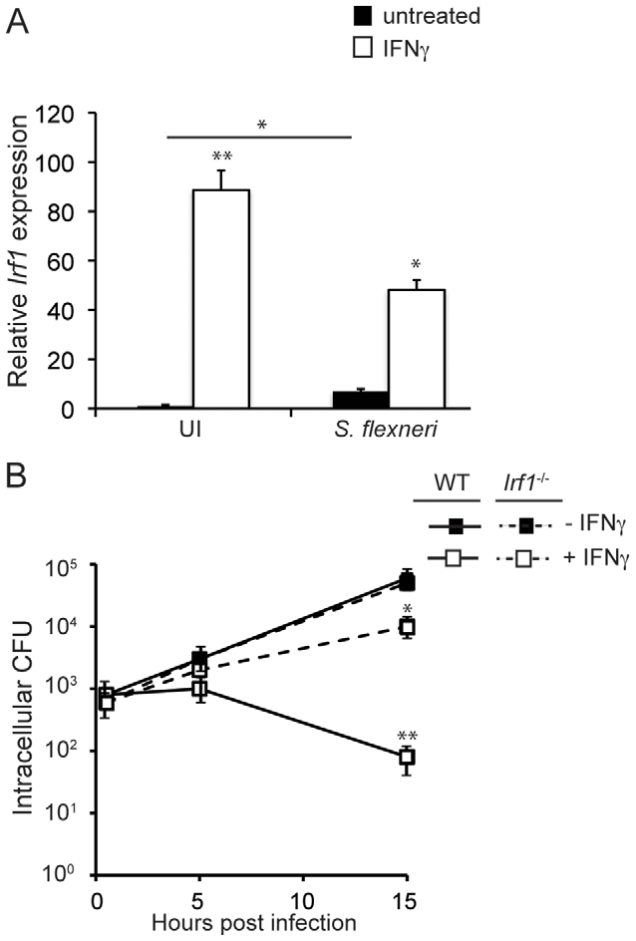
Restriction of *S. flexneri* growth by IFNγ is dependent on IRF1. (A) Analysis of *Irf1* expression by RT-PCR in WT MEFs. MEFs were stimulated with IFNγ for 24 hours or left unstimulated and subsequently infected with WT *S. flexneri* for 6 hours, or left uninfected. *Irf1* transcripts were normalized against host 18S RNA and are shown as means of triplicate samples. (B) Quantification of WT *S. flexneri* CFU in WT or *Irf1* ^−/−^ MEFs. Data are representative of at least 3 independent experiments. Significant statistical differences are indicated as follows: ns, not significant; *, p<0.05; **,p<0.005 (Student's t test). Unless indicated otherwise, noted statistical differences are between unstimulated and IFNγ-stimulated cells for each condition.

Although previous reports had established the ability of IFNγ to inhibit *S. flexneri* growth [9], the cellular mechanisms responsible for this resistance remained completely undefined. One major component downstream of IFNγ signaling that is often required for microbial inhibition by IFNγ is the transcription factor interferon regulatory factor 1 (IRF1), a member of the IRF family of transcription factors, which play broad roles in immunity, oncogenesis, and apoptosis [22], [23], [24]. IFNγ signaling induces the direct transcriptional upregulation of IRF1 and other genes containing gamma-activated-site (GAS) elements in their promoters [25]. IRF1 then translocates to the host cell nucleus, where it binds to interferon stimulated response elements (ISREs) of IFN-stimulated genes (ISGs) and induces a second wave of IFNγ-dependent gene transcription. Although IFNγ-dependent pathogen restriction can occur independently of IRF1, this second wave of transcription induced by IRF1 is often required for IFNγ-mediated growth restriction. To begin to define the cellular pathways and/or gene products that inhibit *S. flexneri* replication in MEFs, we first tested whether IRF1 is induced by IFNγ during infection and whether IRF1 contributes to IFNγ-mediated restriction of this bacterium. We found that IRF1 gene expression was highly induced by IFNγ in uninfected cells, as reported previously (Fig. 2A). Interestingly, *S. flexneri* partially inhibited the ability of IFNγ to upregulate IRF1, even though *S. flexneri* alone induced the upregulation of IRF1 10-fold compared to unstimulated uninfected cells. We next compared *S. flexneri* growth in WT and *Irf1* ^−/−^ MEFs to determine whether IRF1 was required for IFNγ-mediated growth restriction of *S. flexneri*. Although an IFNγ-independent effect of IRF1 on *S. flexneri* replication was not observed, IRF1 significantly contributed to the restriction of *S. flexneri* growth in IFNγ-activated cells by 15 hpi (Fig.2B), confirming the role of IRF1 in the innate immune response to this pathogen.

**Figure 3:**
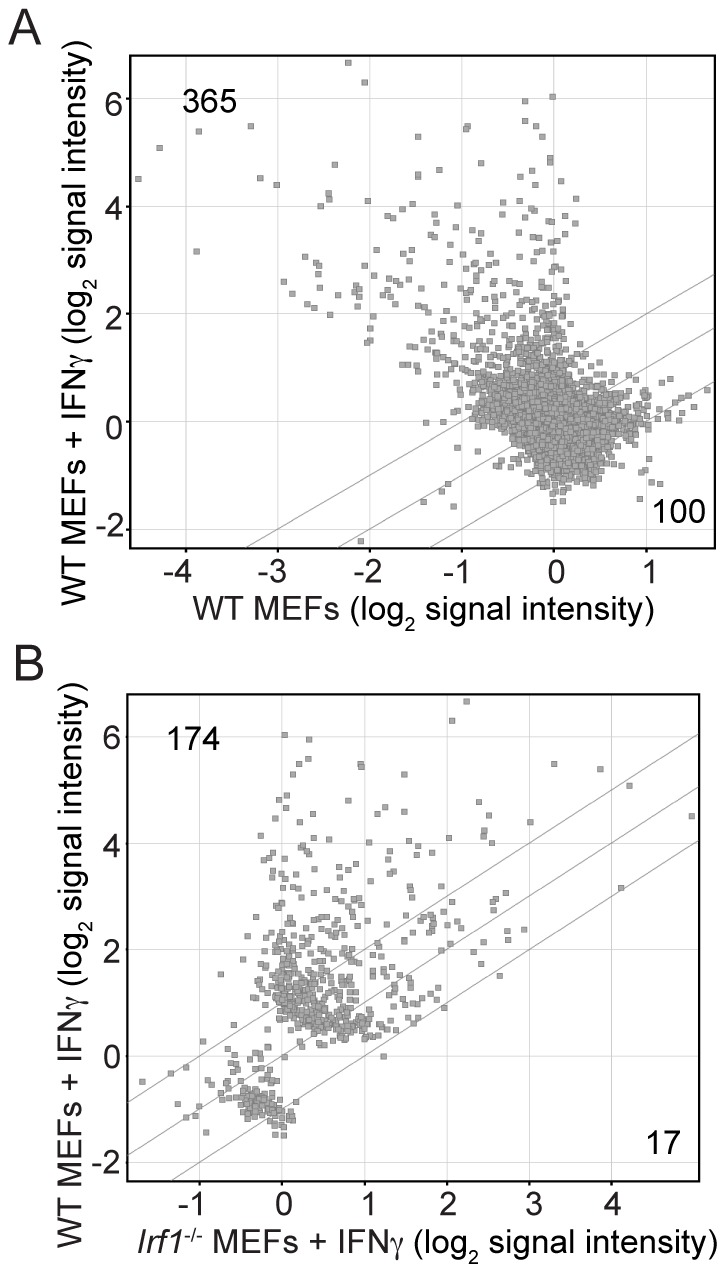
Comparative expression of transcripts from WT and *Irf1* ^−/−^ MEFs. As indicated, cells were stimulated with IFNγ for 18 hours or left unstimulated. All cells were subsequently infected with *S. flexneri* for 6 hours prior to extraction of RNA. (A) Scatter plot of fluorescence intensities of probe sets from unstimulated and IFNγ-stimulated WT MEFs during infection. Dots represent data from individual probe sets and are plotted as normalized, log-2 transformed values. Outer diagonal lines indicate 2-fold differences (p<0.05, ANOVA) in gene expression of a given probe set between different conditions. All probe sets passing minimum threshold conditions during analysis are shown in the plot (see Materials and Methods). (B) Scatter plot of fluorescence intensities of probe sets from IFNγ-stimulated WT and IFNγ-stimulated *Irf1* ^−/−^ MEFs during infection. Probe sets shown in panel B are a subset of the total probe sets shown in panel A and represent only IFNγ-dependent genes that were identified in A. Outer diagonal lines indicate 2-fold differences (p<0.05) in gene expression of a given probe set between different conditions. Microarray data shown are from one experiment out of 2 independent experiments.

Since IRF1 is required for IFNγ-mediated restriction of *S. flexneri*, we hypothesized that an effector mechanism downstream of IRF1-dependent transcription was ultimately required for the inhibition of *S. flexneri* growth. Therefore, we used transcriptional profiling to identify genes that are regulated by IFNγ and dependent on IRF1. Although the identification of IRF1 target genes by microarray analysis has previously been conducted in mouse peritoneal macrophages [26], our goal was to identify genes induced by IFNγ in MEFs specifically during *S. flexneri* infection. We reasoned that IFNγ-inducible gene products that inhibit *S. flexneri* growth might require the cooperation of *S. flexneri*-specific pathogen-associated molecular pattern (PAMP)-mediated signaling in addition to IFNγ for their induction. WT and IRF1^−/−^ MEFs were stimulated with IFNγ or left unstimulated, and all of the cells were subsequently infected with WT *S. flexneri* for 6 hours before total RNA was harvested. Affymetrix mouse whole genome microarrays, representing approximately 20,000 genes, were used to identify IFNγ-dependent, IRF1 target genes. Analysis of the data from unstimulated and IFNγ-stimulated WT MEFs revealed that 365 genes were induced and 100 genes were weakly repressed more than 2-fold by IFNγ during *S. flexneri* infection (Fig. 3A). IFNγ-altered genes included many well-described IFNγ-dependent genes, such as *Stat1*, chemokines including *Cxcl16 and Cxcl9*, anti-viral genes *Oasl1*, *Mx1*, and *Rsad2*, members of the GBP family *Gbp2*, -*3*, and -*6*, as well as several previously uncharacterized genes (). To next identify IFNγ-inducible genes that were dependent on IRF1, we analyzed expression profiles of IFNγ-regulated genes (those identified as altered in Fig. 3A) from IFNγ-stimulated WT cells and IFNγ-stimulated IRF1^−/−^ cells (Fig. 3B). We found that 174, or almost half, of the IFNγ-dependent genes were induced and 17 were repressed by IRF1 (Table S2). This is similar to what was found in peritoneal macrophages in which 387/1,009 IFNγ-induced genes were dependent on IRF1 [26]. The reduction in absolute number of IFNγ-dependent and IFNγ- IRF1-dependent genes identified in our microarray experiments is consistent with previous findings demonstrating that the IFNγ-dependent response in macrophages is more robust than in MEFs [27]. Microarray data from these experiments are available in the ArrayExpress database (www.ebi.ac.uk/arrayexpress) under accession number E-MTAB-713.

### The cytosolic RNA sensing RIG-I/MAVS pathway is required for IFNγ-mediated growth restriction of *S. flexneri*

**Figure 4:**
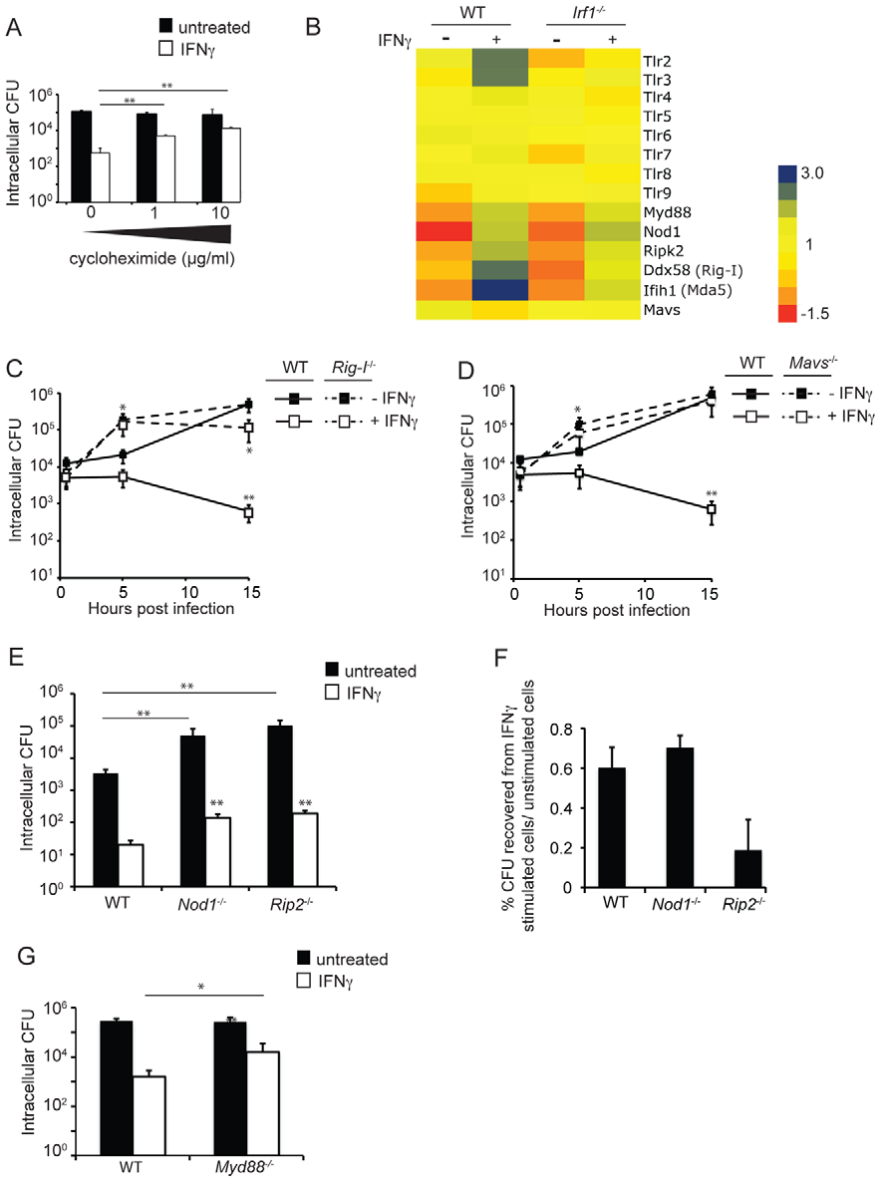
RIG-I and its downstream signaling adaptor MAVS are critical for IFNγ-mediated growth restriction of *S. flexneri*. (A) Host protein synthesis is important for IFNγ-mediated *S. flexneri* growth restriction. MEFs were stimulated with IFNγ for 24 hours or left unstimulated prior to infection. One hour prior to infection, cycloheximide was added at indicated concentrations. Intracellular CFU was determined at 15 hpi. *S. flexneri* invasion into host cells was equivalent under all conditions (not shown). (B) Heat map of intensity signals for selected genes under indicated conditions. Gene colors correspond to log_2_-transformed signal intensities from normalized expression data, indicated by the color key. (C, D) Quantification of *S. flexneri* CFU in indicated MEF cells infected at an MOI of 1∶1. (E, G) Quantification of *S. flexneri* CFU in indicated MEF cells infected at an MOI of 1∶1 for 15 hours. Invasion was equivalent under all conditions (data not shown). (F) Normalization of *S. flexneri* growth inhibition by IFNγ in WT, *Nod1* ^−/−^ and *Rip2* ^−/−^ MEFs. CFU recovered from IFNγ-treated cells for each cell line were normalized against CFU recovered from untreated cells. All data shown are means and standard deviations. Where appropriate, significant statistical differences are indicated as follows: ns, not significant; *, p<0.05; **,p<0.005 (Student's t test). Unless indicated otherwise, noted statistical differences are between unstimulated and IFNγ-stimulated cells for each condition.

Ultimately, we were interested in identifying IFNγ-induced, IRF1-dependent genes capable of blocking *S. flexneri* growth. As we began to think about how to prioritize testing genes identified from the microarray analysis, we first considered our finding that *S. flexneri* growth was not significantly inhibited by IFNγ until at least 5 hpi (Fig. 1A). We hypothesized that this delay in growth restriction might correlate with the amount of time that it would take for full transcriptional or post-transcriptional activation of important antimicrobial genes that require both IFNγ and a signal transmitted to the cell following infection. In support of this hypothesis, we found that blocking host protein synthesis with cycloheximide (CHX), a specific inhibitor of eukaryotic translational elongation, 1 hour prior to the infection (but, importantly, after IFNγ stimulation) blocked the ability of IFNγ to inhibit *S. flexneri* growth in a dose dependent manner (Fig. 4A). Although continued IFNγ-mediated gene induction would also be blocked following CHX treatment, the robust expression of IFNγ-dependent, infection-independent genes would have been strongly upregulated prior to treatment with CHX. These data support the hypothesis that IFNγ-dependent restriction absolutely requires a transcriptional event after infection.

PRRs, which induce changes in gene transcription following the detection of conserved microbial products, would be prime candidates for linking pathogen detection to IFNγ-induced antimicrobial restriction. The Toll-like receptors (TLRs) form one class of PRRs that are expressed at the cell surface or inside of endocytic vesicles and recognize microbial components derived from the extracellular space. Although TLRs are crucial for the detection of most pathogens, the detection of microbial products in the host cytosol requires the action of NLRs and RIG-I-like receptors (RLRs). Cytoplasmic nucleotide-binding oligomerization domain-containing protein 1 (Nod1) and Nod 2 of the NLR family recognize specific structures within peptidoglycan, leading to the recruitment of the adaptor molecule receptor-interacting serine/threonine protein kinase 2 (RIP2) and subsequent RIP2-dependent MAPK and NF-κB activation [28], or RIP2-independent recruitment of autophagsomes at sites of bacterial invasion [29]. The cytoplasmic RLRs RIG-I and melanoma differentiation-associated antigen 5 (MDA5) belong to the phylogenetically conserved DExD/H box family of RNA helicases that recognize various RNA species in the host cytoplasm. Ligand recognition by RIG-I leads to conformational changes that facilitates its association with the downstream signaling adaptor MAVS (also known as VISA, Cardif, IPS-1) at mitochondria and peroxisomes [30], [31]. This interaction results in both the activation of NF-κB and the phosphorylation of IRF-3, leading to the induction of intracellular antimicrobial gene expression and the secretion of type I IFNs, such as IFNβ. To identify a potential PRR that might be required for IFNγ-mediated restriction of *S. flexneri*, we examined the data from our gene expression experiments for PRRs and/or associated signaling adaptor molecules that were identified as IFNγ-dependent, IRF1-dependent genes. Genes encoding several PRRs were highly induced by IFNγ during *S. flexneri* infection and were dependent on IRF1, including the TLRs TLR2 and TLR3 and the RLRs RIG-I (encoded by *Ddx58*) and MDA5 (encoded by *Ifih1*) (Fig. 4B). Since the IFNγ-mediated inhibition of *S. flexneri* occurs in the host cytoplasm, we first focused on the cytosolic RLRs to determine if these molecules play a role in inhibiting *S. flexneri* growth. Additionally, RLRs were interesting because they had not previously been implicated during *S. flexneri* infection. Consistent with our microarray data, it has previously been reported that RIG-I can be induced by IFNγ and is a target gene of IRF1, due to a single IRF1 binding site in its proximal promoter [32], [33], [34]. To determine if RIG-I plays a role in restricting *S. flexneri* during the IFNγ response, we analyzed bacterial growth in WT and *Ddx58* ^−/−^ (referred to as *Rig-I* ^−/−^ for clarity) MEFs. Surprisingly, we found that RIG-I was critical for the ability of IFNγ to inhibit *S. flexneri* growth at 15 hpi (Fig. 4C). Interestingly, RIG-I was also important for IFNγ-independent inhibition of this bacterium at 5 hpi, although it was completely dispensable by 15 hpi. To determine if we could observe a requirement for RIG-I in IFNγ-mediated restriction of a different pathogen that is both restricted by IFNγ and activates the RIG-I pathway, we examined the growth of *L. pneumophila* in IFNγ-stimulated MEFs but failed to observe an IFNγ-dependent effect of RIG-I (Fig. S1).

Although *S. flexneri* has been shown to activate members of both the TLR and NLR families [1], [5], [6], [35], a role for RLRs during *S. flexneri* infection had not previously been demonstrated. Additionally, the requirement for RIG-I in the cell autonomous inhibition of bacterial growth downstream of IFNγ signaling had not been previously described. To determine whether RIG-I-dependent IFNγ-mediated restriction of *S. flexneri* occurs via the canonical RIG-I signaling pathway, we next examined *S. flexneri* growth in WT and *Mavs* ^−/−^ MEFs. MAVS, the downstream signaling adaptor for RIG-I, was critically important for IFNγ-dependent growth restriction of *S. flexneri* (Fig. 4D), demonstrating that RIG-I functions as a signaling molecule acting through its canonically described pathway during *S. flexneri* infection, and not as a MAVS-independent effector of bacterial growth. Interestingly, the requirement for RIG-I and MAVS for *S. flexneri* growth inhibition in non-myeloid cells did not extend to primary macrophages, in which IFNγ-dependent killing appeared to occur independently of both RIG-I and MAVS (Fig. S2).

To determine whether other well-described PRR pathways might similarly play a role in blocking *S. flexneri* replication downstream of IFNγ, we tested the role of the NLR signaling adaptor RIP2, as well as Nod1, which is known to inhibit *S. flexneri* growth independently of RIP2 [29]. *Nod1^−/−^* and *Rip2* ^−/−^ MEFs were more permissive for *S. flexneri* growth compared to WT cells, both in the absence and presence of IFNγ (Fig. 4E). However, in contrast to RIG-I, these proteins were found to be completely dispensable for IFNγ-dependent restriction of this bacterium after normalizing for the IFNγ-independent effects (Fig. 4F). In fact, RIP2^−/−^ MEFs were slightly more efficient than WT MEFs in IFNγ-induced restriction of *S. flexneri* growth (0.2% versus 0.6% CFU recovered in IFNγ-stimulated/unstimulated cells, respectively). In addition to cell-autonomous growth restriction, stimulation of some NLR family members can mediate the induction of the caspase-1 inflammasome in response to microbial infection, leading to the secretion of proinflammatory cytokines, cell death, and the restriction of bacterial replication. However, we found that IFNγ-induced restriction of *S. flexneri* occurred completely independently of caspase-1, suggesting that NLR-mediated inflammasome induction was also not required (data not shown). In contrast to Nod1 and RIP2, the TLR signaling adaptor MyD88 contributed to, but was not required for, the ability of IFNγ to block *S. flexneri* replication (Fig. 4G). Unlike the finding for RIG-I, this result was partially expected, since MyD88 has been shown to be important for TLR-independent IFNγ signaling [36] and for the translocation of IRF1 to the nucleus, at least in myeloid dendritic cells [37]. From our findings, it is unclear whether the role of MyD88 here is solely attributable to one of these previously described functions of MyD88 in IFNγ-mediated signaling or to an alternate mechanism, such as an adaptor of TLR signaling.

**Figure 5:**
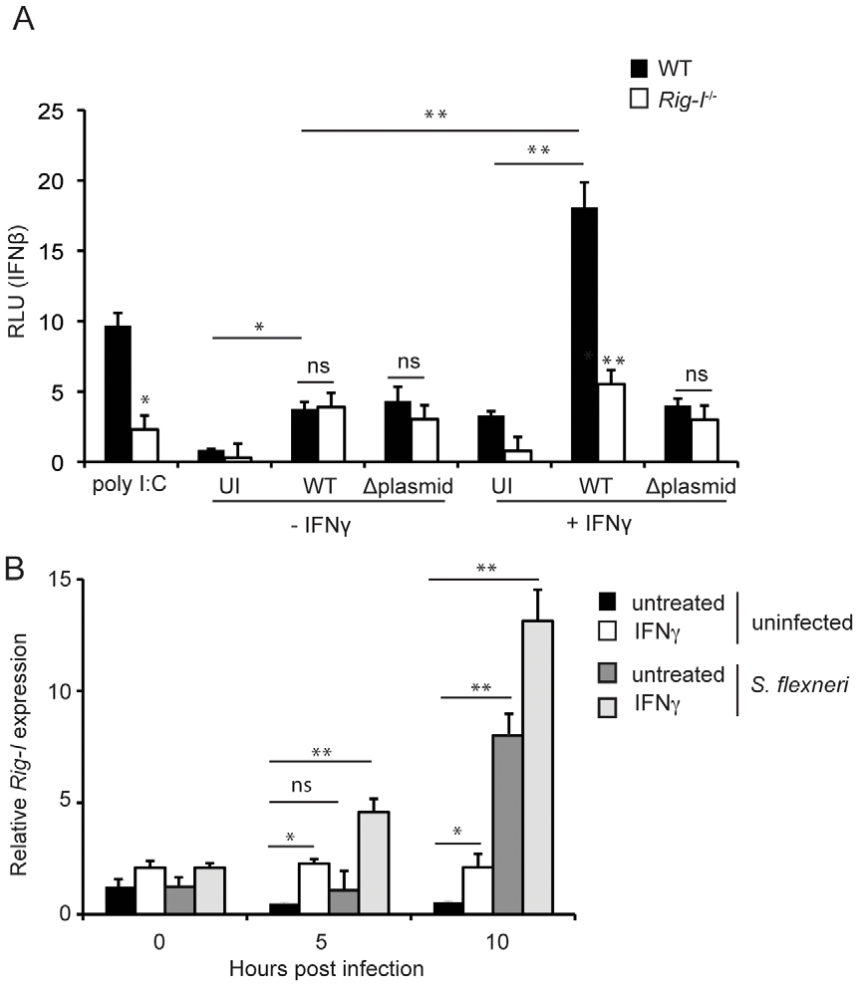
IFNγ potentiates RIG-I-dependent cellular immunity against *S. flexneri*. (A) Quantification of IFNβ secretion by WT and *Rig-I* ^−/−^ MEFs. MEFs were infected for 8 hours with WT or virulence plasmid-cured *S. flexneri*, left uninfected, or transfected for 8 hours with LMW poly (I∶C). Supernatants from the MEFs were then added to L929-ISRE cells, which produce luciferase in response to IFN stimulation. (B) Quantification of *Rig-I* expression in uninfected WT MEFs or MEFs infected with WT *S. flexneri* at an MOI of 1∶1 by quantitative RT-PCR. *Rig-I* levels were normalized to 18S rRNA levels. All data shown are representative of 2 independent experiments. Where appropriate, significant statistical differences are indicated as follows: ns, not significant; *, p<0.05; **,p<0.005 (Student's t test). Unless indicated otherwise, noted statistical differences are between WT and *Rig-I^−/−^* cells for each treatment.

PRR-mediated detection of microbial pathogens, including *S. flexneri*, often results in the transcriptional induction and subsequent secretion of type I IFNs, such as IFNβ, by the host cell [38]. Therefore, the analysis of type I IFN production can be used to assess the cellular immune response to a microbial challenge. To further investigate the role of RIG-I in the cellular immune response to *S. flexneri*, we analyzed IFNβ production from uninfected and *S. flexneri*-infected cells under IFNγ-stimulating conditions. To quantify the bioactivity of IFNβ secreted from infected cells, supernatants from the cells were collected and added to cells that harbor an IFNβ-dependent luciferase reporter (L929-ISRE cells) as described previously [39]. While supernatants from quiescent uninfected WT and *Rig-I* ^−/−^ cells activated the reporter cells with a comparably low efficiency, supernatants from cells transfected with the known RIG-I ligand low molecular weight (LMW) poly (I∶C),a synthetic analog of dsRNA, significantly induced the activation of the reporter cells, and this effect was partially dependent on RIG-I (Fig. 5A). Supernatants from unstimulated cells infected with *S. flexneri* for 8 hours also activated the reporter cells over uninfected cells, but this activation was not dependent on RIG-I. Interestingly, *S. flexneri*-mediated induction of IFNβ secretion was dependent on RIG-I if the MEFs were stimulated with IFNγ prior to the infection. In contrast to WT infection, the IFNβ response to virulence plasmid-cured *S. flexneri* was not dependent on RIG-I, even in the presence of IFNγ (Fig. 5A), suggesting that either access to the cell cytoplasm (where RIG-I is located) or another activity of the Type III secretion system is required for recognition by RIG-I. Collectively, these findings suggest that in unstimulated MEFs, PRRs other than RIG-I dominate the innate immune response to *S. flexneri*, whereas in the presence of IFNγ, RIG-I emerges as an important player in the cellular immune response to this bacterium.

To further characterize the RIG-I-dependent immune response to *S. flexneri*, we also examined RIG-I expression in uninfected and infected cells under stimulating and non-stimulating conditions. Similar to previous reports on other cell types [32], [33], we found that RIG-I is induced 2.5 fold by IFNγ in uninfected MEFs (Fig. 5B). *S. flexneri* infection alone did not induce significant RIG-I expression by 5 hpi, but *S. flexneri*-induced RIG-I expression was apparent by 10 hpi. Despite the lack of induction by *S. flexneri* alone at 5 hpi, IFNγ-induced RIG-I expression was significantly enhanced by the presence of *S. flexneri* at both 5 and 10 hpi. These findings demonstrate that IFNγ and *S. flexneri* synergistically induce RIG-I expression in MEFs, potentially facilitating *S. flexneri* recognition during the IFNγ response.

**Figure 6:**
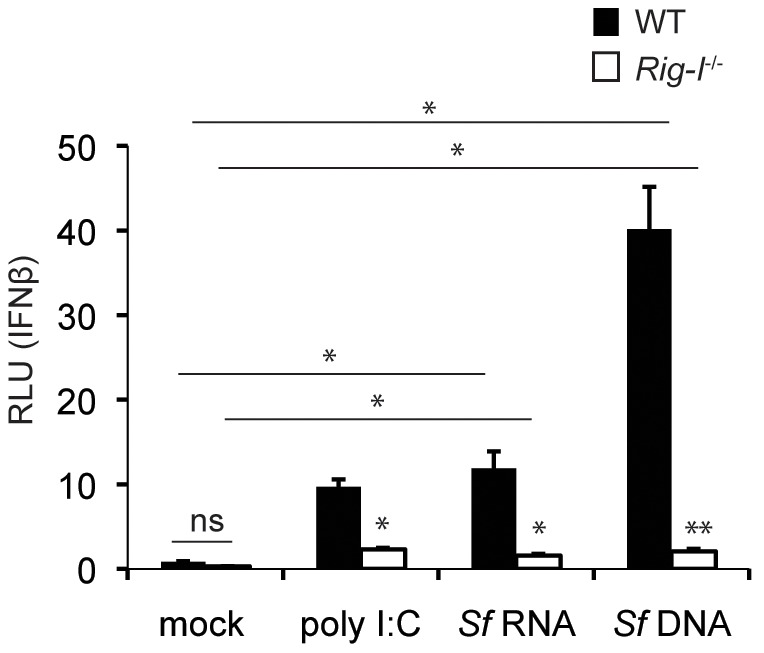
*S. flexneri* nucleic acids are sufficient to induce type I IFN production via RIG-I. Low molecular weight poly (I∶C), extracted *S. flexneri* RNA treated with DNase I, or *S. flexneri* DNA treated with RNase were transfected into MEFs at 0.4 µg ligand/well. Eight hours post transfection, supernatants were added to L929-ISRE cells, which harbor an IFNβ-responsive luciferase reporter. Where appropriate, significant statistical differences are indicated as follows: ns, not significant; *, p<0.05; **,p<0.005 (Student's t test). Unless indicated otherwise, noted statistical differences are between WT and *Rig-I^−/−^* cells for each treatment.

RIG-I contains two N-terminal caspase activation and recruitment domains (CARDs), a DExD/h helicase domain, and a C-terminal repressor domain. In the absence of an activating ligand, the repressor domain maintains RIG-I in an auto-inhibited state in the cell cytoplasm [40]. Therefore, RIG-I signaling does not occur until an activating RNA ligand binds to RIG-I and induces a conformational change that exposes its CARD domain and allows for CARD-CARD interactions with its downstream signaling adaptor MAVS. Due to this mechanism of autoregulation, overexpression of full-length RIG-I is insufficient to induce downstream RIG-I signaling [41]. Therefore, although we found that RIG-I was upregulated in IFNγ-activated cells, the upregulation of RIG-I without an activating ligand is unlikely to be sufficient to induce downstream RIG-I signaling and cannot fully explain the ability of RIG-I to restrict *S. flexneri* growth. Rather, the finding that RIG-I can inhibit *S. flexneri* growth in the presence of IFNγ suggests that *S. flexneri* infection provides or generates a ligand capable of activating RIG-I. Since we found that *S. flexneri* activates RIG-I in the presence of IFNγ, we began to explore the possibility that the ability of *S. flexneri* to activate RIG-I depends upon the presence of *S. flexneri* nucleic acids in the host cytoplasm of activated cells. Although there is debate over the exact nature of the ligand(s) recognized by RIG-I (reviewed in [42]), this molecule is largely thought to recognize cytoplasmic short, double-stranded RNA containing a 5′-triphosphate group (thereby avoiding the recognition of host mRNA, which contains 5′ modifications such as capping). Although RNA species are currently thought to be the only ligands directly recognized by RIG-I, it has been reported that foreign dsDNA introduced into the host cytoplasm can activate RIG-I signaling [43], [44]. More recent reports expanded on these findings to show that RIG-I indirectly recognizes dsDNA introduced into the host cell cytoplasm through the RNA polymerase III-dependent transcription of this DNA into an RNA intermediate that can be recognized by RIG-I [45], [46]. The RNA polymerase III pathway has been shown to be an important component in the recognition of viruses, *L. monocytogenes* [47], and possibly *L. pneumophila*, despite some controversy [45], [48]. To directly test whether *S. flexneri* RNA or DNA is sufficient to induce a RIG-I-dependent cellular response, we extracted *S. flexneri* genomic DNA and total RNA from exponential-phase cultures and then transfected these preparations into WT and *Rig-I* ^−/−^ cells. Importantly, the DNA and RNA preparations were treated with RNase and RNase-free DNase I, respectively, prior to the transfection to eliminate contaminating nucleic acids. Eight hours post-transfection, supernatants from these cells were collected and added to the L929-ISRE IFNβ-dependent luciferase reporter cells. Supernatants from cells transfected with either *S. flexneri* DNA or RNA activated WT cells over basal levels (Fig. 6). Interestingly, the observed activation was dependent on RIG-I in each case, suggesting that both nucleic acid species have the potential to activate a RIG-I-dependent response.

**Figure 7:**
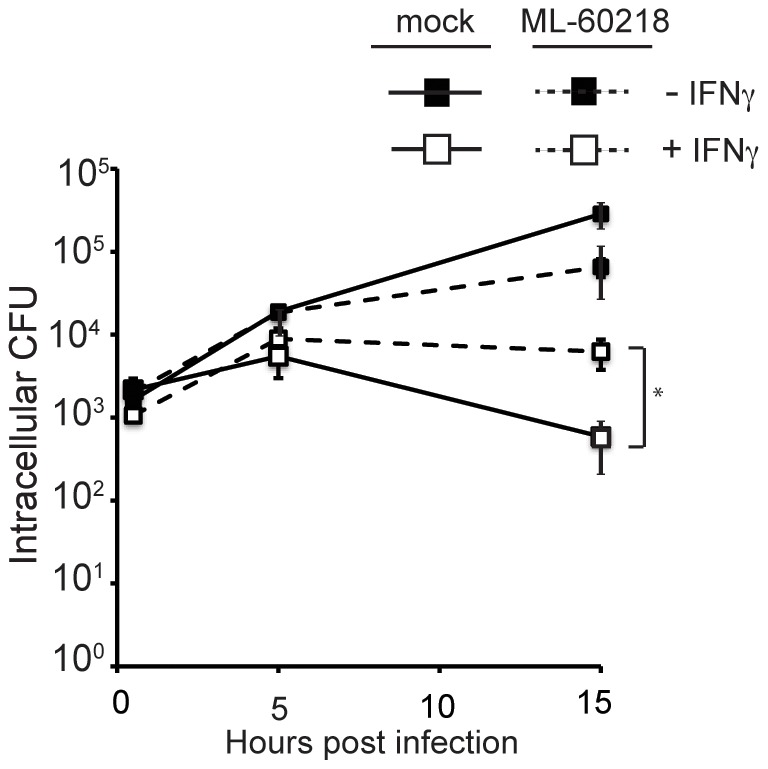
Inhibition of *S. flexneri* by RIG-I is partially dependent on the RNA polymerase III pathway. Quantification of *S. flexneri* CFU in unstimulated or IFNγ-stimulated cells in the presence or absence of the RNA polymerase III inhibitor ML-60218 (20 µM). Data are representative of 3 independent experiments. *, p<0.05 (Student's t test).

Our results suggested that *S. flexneri* DNA has the potential to activate the RIG-I pathway. Currently, the only pathway known to detect bacterial DNA through RIG-I is via the transcription of cytosolic DNA into a 5′-ppp-containing RNA intermediate via the RNA polymerase III pathway [45], [46]. Therefore, we next tested whether host RNA polymerase III is important for IFNγ-mediated suppression of *S. flexneri* growth using an RNA polymerase III-specific inhibitor, ML-60218, described previously [49]. We found that pre-treatment of MEFs with ML-60218 partially blocked the ability of IFNγ to inhibit *S. flexneri* growth by 15 hpi (Fig. 7), potentially suggesting that RNA polymerase III contributes to the detection and subsequent inhibition of this pathogen. It should be noted that significant IFNγ-dependent killing was still observed in the presence of ML-60218, and complete reversal of IFNγ-dependent killing by the inhibitor was not apparent. Interestingly, we did not observe a significant effect of ML-60218 on IFNγ-independent growth restriction, again suggesting that IFNγ enables the functional recognition of intracellular *S. flexneri* by the RIG-I/RNA polymerase III pathway to inhibit *S. flexneri* growth.

**Figure 8:**
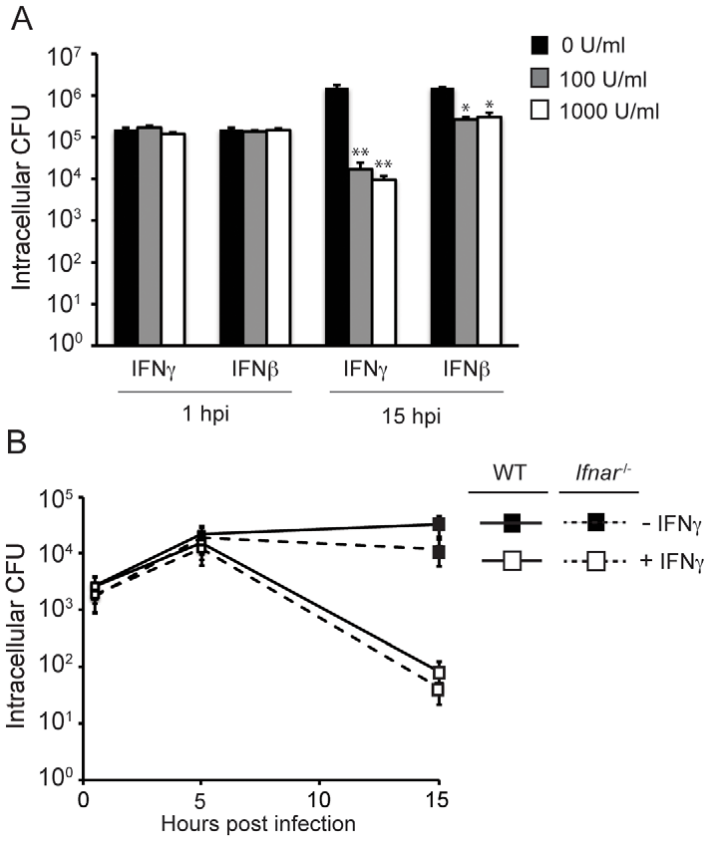
IFNγ-mediated restriction of *S. flexneri* is not dependent on type I IFN signaling. (A) Quantification of *S. flexneri* CFU in WT MEFs stimulated with various concentrations of indicated cytokines. Data are representative of 3 independent experiments. (B) Quantification of *S. flexneri* in WT or *Ifnar* ^−/−^ MEFs stimulated with IFNγ (100 U/ml) for 24 hours or left unstimulated. Significant statistical differences are indicated as follows: ns, not significant; *, p<0.05; **, p<0.005 (Student's t test). Unless indicated otherwise, noted statistical differences are between unstimulated and IFNγ-stimulated cells for each condition.

We next wanted to elucidate the downstream mechanism by which RIG-I enables IFNγ-dependent restriction of *S. flexneri.* RIG-I signaling not only induces the expression of antimicrobial ISGs, but also induces the secretion of type I IFNs, which act in an autocrine manner by binding to the IFN alpha receptor (IFNAR) and sustaining an antiviral state in the host cell. Therefore, we sought to determine whether RIG-I-mediated type I IFN production during infection could explain the role of RIG-I in the inhibition of *S. flexneri* growth. First, we examined the ability of the type I IFN IFNβ to inhibit *S. flexneri* growth in MEFs. At 1 hpi, similar numbers of CFU were recovered from untreated cells and from cells stimulated with IFNγ or IFNβ for 24 hours prior to infection, demonstrating that there was no difference in the invasion of *S. flexneri* into cells under these conditions (Fig. 8A). By 15 hpi, IFNγ potently blocked *S. flexneri* replication by 100-fold, as expected. IFNβ also inhibited *S. flexneri* growth at 15 hpi, however this restriction was much less robust (5-fold growth inhibition), even at very high concentrations. To definitively determine whether type I IFN signaling is important for IFNγ-mediated *S. flexneri* growth restriction, we next examined *S. flexneri* growth in MEFs lacking the type I IFN receptor (*Ifnar* ^−/−^ MEFs), which are completely defective in the ability to respond to type I IFNs. IFNγ-stimulated *Ifnar* ^−/−^ MEFs were significantly more restrictive for *S. flexneri* replication compared to WT MEFs. These findings demonstrate that while type I IFNs are capable of inhibiting *S. flexneri* growth (albeit to a lesser extent compared to IFNγ), they are dispensable for IFNγ-mediated growth restriction of this bacterium. Together these findings suggest that the inhibition of *S. flexneri* by IFNγ occurs due a type I IFN secretion-independent mechanism following RIG-I signaling.

**Figure 9:**
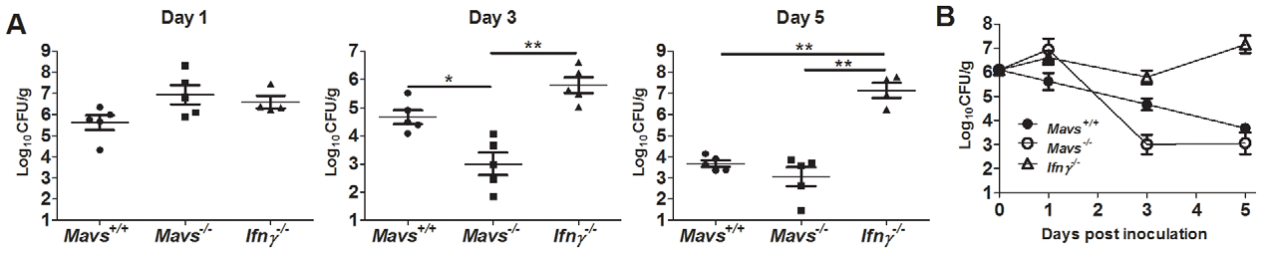
MAVS appears to control *S. flexneri* growth at early timepoints but is not required for clearance *in vivo*. (A) Quantification of *S. flexneri* load per gram of lung tissues. *IFNγ* ^−/−^, *Mavs* ^−/−^, and *Mavs* ^+/+^ were infected intranasally with 3×10^5^ CFU *S. flexneri*. On indicated days post-infection, lungs were harvested and plated for bacterial CFU enumeration. Statistical differences are indicated as follows:* p<0.05, **, p<0.005 (one-way ANOVA). (B) Quantification of *S. flexneri* load per gram of lung tissues over time. Data are representative of 3 independent experiments.

To further investigate the importance of the IFNγ/RIG-I/MAVS pathway in protection against *S. flexneri in vivo*, we analyzed *S. flexneri* growth in *IFNγ* ^−/−^, *Mavs* ^−/−^, and *Mavs* ^+/+^ mice (*Rig-I* ^−/−^ mice are embryonic lethal and were therefore not included). Mice were infected intranasally with 3×10^5^ *S. flexneri,* and the lungs were harvested at 4 hours and 1, 3, and 5 days post-infection. *Mavs* ^−/−^ mice and *IFNγ* ^−/−^ mice harbored bacterial burdens that were nearly 1 log greater than those observed in *Mavs*-sufficient mice by day 1 post-infection (Fig. 9). Although this difference was not statistically significant among groups, this trend towards greater bacterial burden in *Mavs* ^−/−^ mice on day 1 post-infection was consistently observed in 3 independent experiments. Surprisingly, while the burdens in *IFNγ* ^−/−^ mice continued to increase through day 5 (consistent with previous reports, [9]), bacterial burdens in *Mavs* ^−/−^ mice dramatically declined by day 3 by several logs, to levels below those seen in MAVS-sufficient mice. Although the role of the MAVS pathway during *S. flexneri* infection remains to be fully elucidated, these findings suggest that early during infection the MAVS pathway plays a small role in the inhibition of *S. flexneri* replication in vivo. Collectively, the findings presented here implicate the RIG-I/MAVS immunosurveillance pathway as an important component in IFNγ-mediated cell autonomous restriction of a cytosolic bacterial pathogen.

## Discussion

During an infection, host cells must simultaneously respond to multiple signals, including both host-derived factors and microbe-derived factors in order to exert the appropriate immune response. Here we describe the unexpected mutual requirement for both host-derived IFNγ and infection-dependent stimulation of an RNA-sensing pathway in order to mediate the inhibition of a cytosolic bacterial pathogen. For years, cytosolic RNA-sensing pathways were thought to respond only to viral pathogens. More recently, the microbes capable of stimulating RNA-sensing pathways has expanded to include the recognition of bacterial pathogens as well, including *L. pneumophila* by RIG-I and MDA5 [48], *L. monocytogenes* by the RLR laboratory of genetics and physiology 2 (LGP2) [47], and now also *S. flexneri* by RIG-I. We found that RIG-I is key to the recognition and subsequent elimination of *S. flexneri* during the IFNγ response, potentially through the recognition of both *S. flexneri* RNA and an RNA polymerase III-transcribed RNA intermediate derived from *S. flexneri* DNA. Together these findings implicate RNA-sensing pathways as critical players in the IFNγ-mediated cell autonomous restriction of a cytosolic bacterial pathogen.

Similar studies on other cytoplasmic pathogens have been performed, although these studies have largely been conducted in macrophages. In macrophages, IFNγ prevents the escape of *L. monocytogenes* and *F. novicida* to the host cytoplasm, while *F. tularensis* is not inhibited by IFNγ until the bacteria have reached the host cytoplasm [15], [16], [17]. Although macrophages are often the primary mediators of IFNγ-inducible killing, complete clearance of *S. flexneri* must result from intracellular resistance mechanisms induced at the site of *S. flexneri* replication, in the epithelial cell layer. Therefore, in these studies, we used non-myeloid MEFs as a model for epithelial cells to understand how IFNγ enables the host to control, and finally clear, an infection with this pathogen. We found that IFNγ inhibits the growth of *S. flexneri* in MEFs at the step of cytosolic replication, and not at the earlier stages of entry and vacuolar escape (Fig. 1). Taken together, these reports highlight the complexity of the IFNγ response in terms of its ability to recognize and inhibit different pathogens—even different cytosolic bacterial pathogens—through distinct mechanisms and at different locations. In the case of *S. flexneri*, we hypothesize that the >5 hour delay in the ability of IFNγ-activated cells to block *S. flexneri* replication corresponds to the recognition of the bacterium by RIG-I and subsequent induction of antimicrobial mechanisms. It is possible that during human infection this delay is sufficient for *S. flexneri* to begin its cycle of replication and cell-to-cell dissemination prior to being killed by IFNγ-induced mechanisms, further pushing the host-pathogen balance in favor of the bacteria. Indeed, IFNγ added to cells at the time of the infection had no effect on *S. flexneri* growth by 15 hpi (data not shown); it is possible that *S. flexneri* is capable of actively interfering with IFNγ-mediated signaling and can establish a productive infection if the cells are not stimulated prior to the infection. Alternatively, this delay could be a reflection of the time that it would take for IFNγ-dependent IRF1 induction and subsequent IRF1-, RIG-I- mediated immune mechanisms to be induced. These possibilities certainly warrant further investigation.

Synergy between IFNγ and PRRs has long been appreciated as a crucial component of innate immunity. In some cases, IFNγ effectively lowers the concentration of PAMP ligands required to affect downstream PRR-dependent gene regulation. In other cases, IFNγ priming is absolutely required for downstream PRR-dependent gene induction [50]. Conversely, PRR signaling can also enhance IFNγ-dependent gene induction. In the case of RIG-I, it has been reported that RIG-I can potentiate IFNγ-induced expression of the chemokines CXCL9–11, although the functional consequences of this finding on microbial infection has not been explored [51], [52]. We extended these findings by identifying a role for RIG-I in mediating IFNγ-dependent cell autonomous resistance to a cytosolic bacterial pathogen. The findings presented here raise two fundamentally important but distinct questions. How might RIG-I link IFNγ signaling to inhibition of *S. flexneri* growth? Secondly, how does IFNγ potentiate the recognition of *S. flexneri* by RIG-I? To first address how RIG-I links IFNγ signaling with inhibition of *S. flexneri* growth, we considered that RIG-I is important for the expression of important antimicrobial ISGs induced by IFNγ during *S. flexneri* infection. In support of this hypothesis, our preliminary data suggest that ISG expression is altered by RIG-I downstream of IFNγ during infection (data not shown). Although previous reports have demonstrated that PRRs can inhibit microbial growth independently of their canonical downstream signaling adaptors [29], the absolute requirement for MAVS in the inhibition of *S. flexneri* by IFNγ suggests that RIG-I functions as a signaling molecule and not through a MAVS-independent mechanism. Furthermore, our results demonstrate that the RIG-I-dependent effect occurs in a mechanism independent of type I IFN signaling. Therefore, overall we favor a model in which RIG-I modulates ISG expression or other cell-intrinsic MAVS-dependent antimicrobial mechanisms to inhibit *S. flexneri* growth during the IFNγ response. What remains unclear from our findings is whether RIG-I acts upstream or downstream of IRF1. An alternative, and equally valid, model to the one presented here is that the cell upregulates RIG-I upon sensing of *S. flexneri* infection, allowing for the activation of RIG-I and subsequent gene transcription through IRF1, which will have been previously upregulated by IFNγ.

Secondly, to address how IFNγ potentiates a RIG-I-dependent response against *S. flexneri*, we considered several findings. We found that RIG-I expression was induced by IFNγ but was not induced by *S. flexneri* in the absence of IFNγ at early timepoints. Because RIG-I failed to be upregulated by *S. flexneri* early during infection, it is possible that greater RIG-I expression in the presence of IFNγ accounts for the ability of RIG-I to ultimately restrict *S. flexneri* growth. While upregulation of RIG-I alone may not fully activate its downstream signaling cascade, the stimulation of a greater number of RIG-I molecules might be necessary to overcome a potential evasion of RIG-I signaling by *S. flexneri*. However, we also considered an alternative possibility, in which IFNγ-mediated, RIG-I-independent effector mechanisms induce some amount of lysis or damage to a small number of *S. flexneri* early during infection, releasing bacterial nucleic acids into the cytoplasm that lead to the activation of RIG-I. This model, or an alternate model requiring more than the simple upregulation of RIG-I, is more consistent with our findings, since IFNβ was a weak inhibitor of *S. flexneri* growth (compared to IFNγ), despite its demonstrated ability to upregulate RIG-I expression.

We found that *S. flexneri* RNA and genomic DNA can each induce a RIG-I dependent immune response in MEFs (Fig. 6), however the actual ligand for RIG-I during *S. flexneri* infection remains to be identified. Whether *S. flexneri* DNA or RNA actually reach the cytoplasm during infection is not known. The finding that the RNA polymerase III pathway partially contributed to the inhibitory effect of IFNγ on *S. flexneri* (Fig. 7) suggests that *S. flexneri* DNA accesses the cytoplasm and is involved in the IFNγ-mediated immune response during infection. It remains to be determined whether *S. flexneri* DNA reaches the cytoplasm by being shed during normal bacterial replication, bacterial cell lysis, direct translocation of DNA into the host cytoplasm, or other alternate mechanisms. The RNA polymerase III pathway appeared to only partially contribute to the inhibition of *S. flexneri* replication, however, supporting the idea that *S. flexneri* RNA also potentially activates RIG-I during infection. In support of this hypothesis, activation of RIG-I signaling by bacterial RNA has previously been demonstrated for *L. pneumophila* [48]. Finally, in the case of *S. flexneri* infection, we cannot discount the possibility that the infection induces the recognition of host-derived RIG-I ligands through the disruption of cellular processes or damage to host organelles. In fact, in response to viral infections, host nuclease RNase L can produce small RNAs from host RNA that can serve as RIG-I ligands [53].

The RNA polymerase III pathway has been shown to be activated by certain viral and AT-rich DNA [46], however its role in antibacterial immunity remains to be fully elucidated. Using the RNA polymerase III inhibitor ML-60218, Chiu, et. al reported that *L. pneumophila* activates type I IFN production via the RNA polymerase III/RIG-I pathway in RAW macrophages, resulting in inhibition of bacterial growth [45]. A counter report by Monroe, et al. showed that the RNA polymerase III pathway failed to affect *L. pneumophila* replication in bone marrow-derived macrophages (BMDMs) [48], calling into question the role of the RNA polymerase III pathway during *L. pneumophila* infection. More recently, it was demonstrated that type I IFN production induced by *L. monocytogenes* in BMDM is dependent on RNA polymerase III [47]. Here, we report that the ML-60218 inhibitor partially relieved IFNγ-dependent killing of *S. flexneri* in MEFs, suggesting that RNA polymerase III may play a role in this pathway. Despite this finding, future studies using alternate inhibitors of this pathway and/or additional techniques will be needed to firmly establish the role of the RNA polymerase III/RIG-I pathway in immunity against *S. flexneri*.

Interestingly, not all PRRs stimulated by *S. flexneri* exhibited IFNγ-dependent effects on *S. flexneri* replication. It is possible that NLR ligands are simply equally available to NLRs in both the absence and presence of IFNγ, whereas RIG-I ligands are significantly more accessible under IFNγ stimulation, as discussed above. Although MAVS was crucial for *S. flexneri* restriction by IFNγ, we did observe some IFNγ-dependent, RIG-I-independent effects on growth inhibition (Fig. 4C), suggesting that MDA5 or other MAVS-dependent host factors might also be involved in *S. flexneri* recognition during the IFNγ response. Finally, it will be interesting to investigate the role of MyD88 in the IFNγ response against *S. flexneri*. One possibility is that TLR2, which we identified as an IFNγ-inducible, IRF1-dependent gene, mediates the MyD88-dependent effect. However, the strict requirement for MAVS in the IFNγ response suggests an alternate role for MyD88, such as the MyD88-dependent translocation of IRF1 into the nucleus, which has been shown to occur in myeloid dendritic cells [37]. Collectively, we favor a model in which early in infection NOD-like receptors inhibit the initial growth of *S. flexneri* in the epithelial cell layer, but at later times (following the recruitment of IFNγ-producing NK cells and IFNγ secretion at the site of infection) RIG-I becomes a crucial component in the ability of the host to clear *S. flexneri* infection.

In vivo, the MAVS pathways appeared to play only a minor role in inhibiting *S. flexneri* growth, contributing to protection only at very early timepoints. One explanation is that IFNγ-dependent control of *S. flexneri* occurs in non-myeloid cells (such as epithelial cells) early during infection, while at later timepoints IFNγ-activated macrophages dominate the IFNγ-dependent response; consistent with this hypothesis, we found that neither RIG-I nor MAVS is important for IFNγ-dependent killing of *S. flexneri* in primary macrophages (Fig. S2). However, considering that even unactivated macrophages are non-permissive for *S. flexneri* replication, the importance of macrophages in the IFNγ response awaits further investigation. Equally valid is the possibility that RIG-I/MAVS-dependent effector mechanisms are activated prior to other intracellular resistance mechanisms, making this pathway important only until other pathways have been activated, at which time they become dispensable. This hypothesis is consistent with the finding that bacterial burdens in *Mavs* ^−/−^ mice decreased dramatically by Day 3, suggesting that other antimicrobial pathways are in place and that these pathways can compensate for MAVS in its absence.

Collectively these experiments deepen our understanding of the many pathways used by host cells to inhibit infections with cytosolic bacterial pathogens. While the discovery of RIG-I as a mediator of the antimicrobial host response downstream of IFNγ and IRF1 is exciting, ultimately it will be interesting to identify the downstream effector mechanisms that inhibit or kill *S. flexneri* in host cells. Studies on other cytosolic bacterial pathogens such as *L. monocytogenes* have found that IFNγ-mediated growth restriction depends upon the induction of RNS and ROS delivered to nascent pathogen-containing vacuoles [17], [18]. While these pathways are established resistance mechanisms in macrophages and other phagocytic cells, these pathways are thought to play a relatively minor role in IFNγ-dependent immunity in non-myeloid cells. Indeed, our preliminary results suggest that RIG-I-dependent killing of *S. flexneri* in IFNγ-activated MEFs occurs independently of ROS (unpublished data). Recent advances in the elucidation and discovery of IFNγ-dependent antimicrobial pathways in non-myeloid cells such as MEFs have led to the characterization of a multitude of protein families and pathways capable of exerting cell autonomous resistance against microbial pathogens, such as the p47 GTPase and p65 GTPase families (reviewed in [54], [55]). Due to the abundance of pathways induced by IFNγ, the microarray experiments described in this paper will provide a crucial starting point for unraveling the complexity of the IFNγ response against this pathogen. Which of these described, or previously undescribed, genes are important for blocking *S. flexneri* replication in host cells awaits further investigation.

## Materials and Methods

### Ethics statement

This study was carried out in strict accordance with the recommendations in the Guide for the Care and Use of Laboratory Animals of the National Institutes of Health. The protocol was approved by Harvard's Animal Care and Use Committee.

### Bacterial strains

*Shigella flexneri* serovar 2a WT strain 2457T [56] and WT strain 2457T transformed with p-GFPmut2 [57], virulence-plasmid cured strain BS103 [58], and MGB283 (*icsA*) on the 2457T background were described previously. *Legionella pneumophila* WT serogroup 1 strain was used [59].

### Cell culture

WT and *Irf1* ^−/−^ mouse embryonic fibroblasts (MEFs) were isolated from day 12.5–14.5 embryos. Cell lines that were not generated in our lab were obtained as follows: *Rig-I* ^−/−^ and matched WT MEFs (L. Gherke, Harvard Medical School); *Nod1* ^−/−^, *Rip2* ^−/−^ and matched WT MEFs (D. Philpott, University of Toronto); *Myd88* ^−/−^ MEFs (E. Kurt-Jones, University of Massachusetts), *Mavs* ^−/−^ MEFs, WT, *Rig-I* ^−/−^, and *Mavs* ^−/−^ immortalized primary BMMs (J. Kagan, Harvard Medical School); *Ifnar* ^−/−^ MEFs (B. Burleigh, Harvard School of Public Health); L929-ISRE fibroblasts (B. Beutler, The Scripps Research Institute). Cells were grown in DMEM (Invitrogen) supplemented with 10% FBS, 1× non-essential amino acids, 1× sodium pyruvate, 100 µM streptomycin, and 100 U/ml penicillin.

### IFNγ stimulation and in vitro infections

Unless indicated otherwise, 100 U/ml recombinant mouse IFNγ (Chemicon International) was added to cells 24 hours prior to infection and was maintained throughout the infection. Cells were infected with *S. flexneri* by centrifuging exponential phase bacteria diluted in PBS onto semi-confluent monolayers of cells at an MOI of 1∶1 at 700×g for 10 minutes. The cells were subsequently incubated for 20 minutes at 37°C and 5% CO_2_, washed 3 times with PBS, and resuspended in media containing gentamicin (25 µg/ml) to kill extracellular bacteria. To assess intracellular bacterial number, the cells were then incubated for indicated amounts of time in media containing gentamicin, washed 3 times with PBS, and lysed in 0.1% sodium deoxycholate/PBS. Cell lysates were then plated on tryptic soy agar (TSA) plates, and CFU were counted after overnight incubation at 37°C. For *L. pneumophila* experiments, *L. pneumophila* were grown on charcoal yeast extract (CYE) agar for 2 days prior to infections. Heavy patch cultures were subsequently resuspended in PBS and centrifuged onto semi-confluent monolayers of cells at an MOI of 30∶1 at 700×g for 10 minutes. The cells were subsequently incubated for 50 minutes at 37°C and 5% CO_2_, washed 3 times with PBS, and resuspended in media containing gentamicin (25 µg/ml) to kill any extracellular bacteria. To assess intracellular bacterial load, cell monolayers were lysed in sterile water, plated on CYE agar plates, and incubated at 37°C for 48 hours prior to CFU enumeration.

### Mice and in vivo infections

B6;129-Mavstm1Zjc/J (*Mavs* ^+/−^) and B6.129S7-Ifnγtm1Ts/J (IFNγ^−/−^) mice were obtained from Jackson Laboratories (Bar Harbor) and bred in specific pathogen-free breeding rooms at Harvard Medical School. *Mavs* ^+/−^ mice were bred with each other, and *Mavs* ^+/+^ and *Mavs* ^−/−^ littermates were used in the experiments. For infections, *S. flexneri* was subcultured from an overnight culture to exponential phase (OD_600_ = 0.4–0.6), and diluted with PBS to the appropriate concentration prior to inoculation. Numbers of bacteria per inoculums were confirmed by plating serial dilutions of the inoculum. For inoculation, 6–8 week-old mice were lightly sedated with 5% isoflurane (Vedco, Inc) in oxygen and inoculated by pipetting 40 µL PBS containing 2.5×10^5^ CFU of bacteria onto the external nares. For quantification of bacteria numbers, mice were sacrificed via CO_2_ inhalation and lungs were excised, homogenized in 2 mL PBS, serially diluted and plated onto TSA plates containing Congo red (0.01%). Colonies were counted after incubation at 37°C for 12–18 hours. The lower limit of detection was 20 CFU.

### Immunofluorescence microscopy

Cells were grown and infected on glass cover slips for indicated amounts of time. Cells were then washed, fixed in 4% paraformaldehyde for 10 min, washed in PBS, and permeabilized with 0.1% Triton-X for 10 min. Actin was visualized by staining with an Alexa Fluor 647-conjugated phalloidin (Invitrogen) according to the manufacturer's directions.

### Microarrays

MEFs were stimulated with IFNγ for 18 hours or left unstimulated. All cells were subsequently infected at an MOI of 1∶1 for 6 hours, a time when the transcription of genes that require both IFNγ and molecular sensing of *S. flexneri* for their regulation would be altered. Total RNA was harvested at 6 hpi using the RNeasy kit (Qiagen) and subsequently treated with DNase I. Generation of cDNA, cRNA, biotinylation, fragmentation, and hybridization to Affymetrix mouse whole genome 430 2.0 arrays were performed at the Harvard Biopolymers Facility. The array was repeated two times using biological replicates. Data from each of the 4 samples in one array were first normalized using the MAS 5.0 algorithm using Gene Spring GX software. Next, probe sets in which 0/4 samples exhibited expression between 20% and 100% were dropped from further analysis. Out of the 45,101 probe sets represented on the arrays, 37,569 probe sets had at least one sample with a value within the cut-off threshold and were kept for analysis.

### Quantitative RT-PCR

Total RNA from MEFs was harvested 6 hpi using the RNeasy kit (Qiagen) according to the manufacturer's directions. RNA samples were treated with DNase I prior to reverse transcription and amplification with SYBR Green One-Step Quantitative RT-PCR kit (Qiagen). Transcript levels were normalized to 18S rRNA. The following primer sequences were used: *Irf1*: F, 5′-TTAGCCCGGACACTTTCTCTGATGG-3′ and R, 5′-GTCCCCTCGAGGGCTGTCAATCTCT-3′; *Rig-i:* F, 5′-ATTGTCGGCGTCCACAAAG-3′ and R, 5′-GTGCATCGTTGTATTTCCGCA-3′; *18S rRNA*: F, 5′-CATTCGAACGTCTGCCCTATC-3′ and R, 5′-CCTGCTGCCTTCCTTGGA-3′.

### Transfection of *S. flexneri* nucleic acids and Type I IFN secretion assays

Total bacterial genomic DNA was isolated using the DNeasy kit (Qiagen) in conjunction with RNaseA treatment (Qiagen) at 100 mg/ml for 2 min. Total bacterial RNA was isolated using RNAprotect Bacterial Reagent (Qiagen) and the RNeasy kit (Qiagen); isolated RNA was subsequently treated with DNAase I at 10 U/ml for 10 minutes at 37°C and re-purified using the RNeasy kit. Transfections of isolated bacterial DNA, RNA, or low molecular weight poly (I∶C) (Invivogen) into MEFs were performed by mixing indicated nucleic acids with DMEM and Attractene (Qiagen) to a final ratio of 0.25 µg nucleic acid/µl Attractene and incubated for 20 minutes. Lipid-ligand complexes were added to cells at a quantity of 0.4 µg ligand/well of a 24 well plate. Eight hours post-transfection or post-infection with *S. flexneri*, supernatants were collected and overlaid onto pre-seeded L929-ISRE cells, which harbor an IFNβ-dependent luciferase reporter, for 4 hours. Luciferase expression from L929-ISRE cells was quantified using Bright Glo (Promega) according to the manufacturer's directions.

### Inhibitors

Where indicated, cycloheximide was added to cells 2 hours prior to infection at indicated concentrations and was maintained throughout the experiment. The RNA polymerase III inhibitor ML-60218 (Calbiochem) was added to cells 2 hours prior to infection at 20 µM and maintained throughout the experiment.

### Statistical analysis

As indicated, a two-tailed Student's *t* test for paired samples or a one-way ANOVA was used to determine statistical significance. A *p* value<0.05 was considered statistically significant.

### Accession numbers/ID numbers

Microarray data are available in the ArrayExpress database (www.ebi.ac.uk/arrayexpress) under accession number E-MTAB-713. Entrez gene ID numbers for genes mentioned in the text are as follows: *Irf1*: 16362; *Rig-I*: 230073; *Mavs*: 228607; *Nod1*: 107607; *Rip2*: 192656; *Myd88*: 17874; *Ifnar*: 15975.

## Supporting Information

Figure S1 **IFNγ-dependent restriction of** ***L. pneumophila*** **occurs independently of RIG-I in MEFs.** To determine if we could observe a requirement for RIG-I during the IFNγ-mediated restriction of a pathogen other than *S. flexneri*, we examined the growth of *L. pneumophila*, a pathogen that is both restricted by IFNγ and activates the RIG-I pathway, in MEFs. WT and *Rig-I^−/−^* MEFs were infected at an MOI of 30∶1, and the intracellular growth of *L. pneumophila* was analyzed at 15 hpi. *L. pneumophila* uptake into host cells was equivalent under all conditions (data not shown). As had been reported previously in macrophages [48], we observed that WT *L. pneumophila* replication was inhibited by RIG-I in unstimulated MEFs and that *L. pneumophila* was restricted by IFNγ (A). However, normalization of CFU recovered from IFNγ-treated cells against CFU recovered from untreated cells showed that growth inhibition of this bacterium by IFNγ occurred independently of RIG-I (B). All data shown are means and standard deviations. Where appropriate, significant statistical differences are indicated as follows: ns, not significant; *, p<0.05; **, p<0.005 (Student's t test). Unless indicated otherwise, noted statistical differences are between unstimulated and IFNγ-stimulated cells for each condition.
(TIF)

Figure S2 **RIG-I and MAVS are dispensable for IFNγ-dependent restriction of** ***S. flexneri*** **in primary BMMs.** Quantification of *S. flexneri* CFU in WT, *Rig^−I^* ^−^, and *Mavs^−/−^* BMMs infected at an MOI of 1∶1 for indicated amounts of time. All data shown are means and standard deviations. Where appropriate, significant statistical differences are indicated as follows: ns, not significant; *, p<0.05; **, p<0.005 (Student's t test). Unless indicated otherwise, noted statistical differences are between unstimulated and IFNγ-stimulated cells for each condition.
(TIF)

Table S1 **List of genes regulated by IFNγ during** ***S. flexneri*** **infection.**
(XLS)

Table S2 **List of IFNγ-regulated, IRF1-dependent genes during** ***S. flexneri*** **infection.**
(XLS)
