# Supplementary figures and images for: IFNγ Inhibits the Cytosolic Replication of Shigella flexneri via the Cytoplasmic RNA Sensor RIG-I
Source: PLoS Pathog. 2012 Aug 9;8(8):e1002809. doi: 10.1371/journal.ppat.1002809 (PMC3415441; doi:10.1371/journal.ppat.1002809)

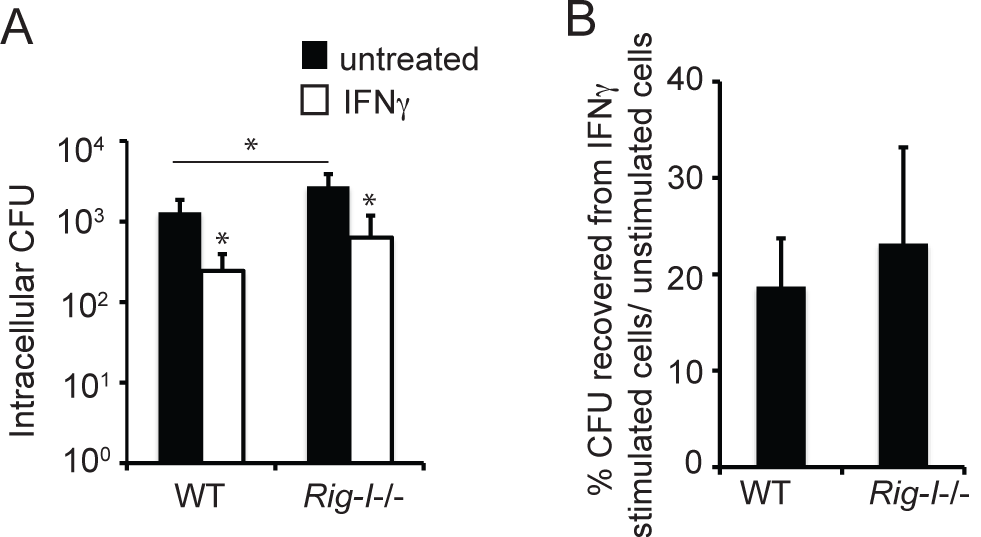

Supplement: Figure S1 — IFNγ-dependent restriction of L. pneumophila occurs independently of RIG-I in MEFs. To determine if we could observe a requirement for RIG-I during the IFNγ-mediated restriction of a pathogen other than S. flexneri, we examined the growth of L. pneumophila, a pathogen that is both restricted by IFNγ and activates the RIG-I pathway, in MEFs. WT and Rig-I−/− MEFs were infected at an MOI of 30∶1, and the intracellular growth of L. pneumophila was analyzed at 15 hpi. L. pneumophila uptake into host cells was equivalent under all conditions (data not shown). As had been reported previously in macrophages [48], we observed that WT L. pneumophila replication was inhibited by RIG-I in unstimulated MEFs and that L. pneumophila was restricted by IFNγ (A). However, normalization of CFU recovered from IFNγ-treated cells against CFU recovered from untreated cells showed that growth inhibition of this bacterium by IFNγ occurred independently of RIG-I (B). All data shown are means and standard deviations. Where appropriate, significant statistical differences are indicated as follows: ns, not significant; *, p<0.05; **, p<0.005 (Student's t test). Unless indicated otherwise, noted statistical differences are between unstimulated and IFNγ-stimulated cells for each condition. (TIF) [file ppat.1002809.s001.tif]

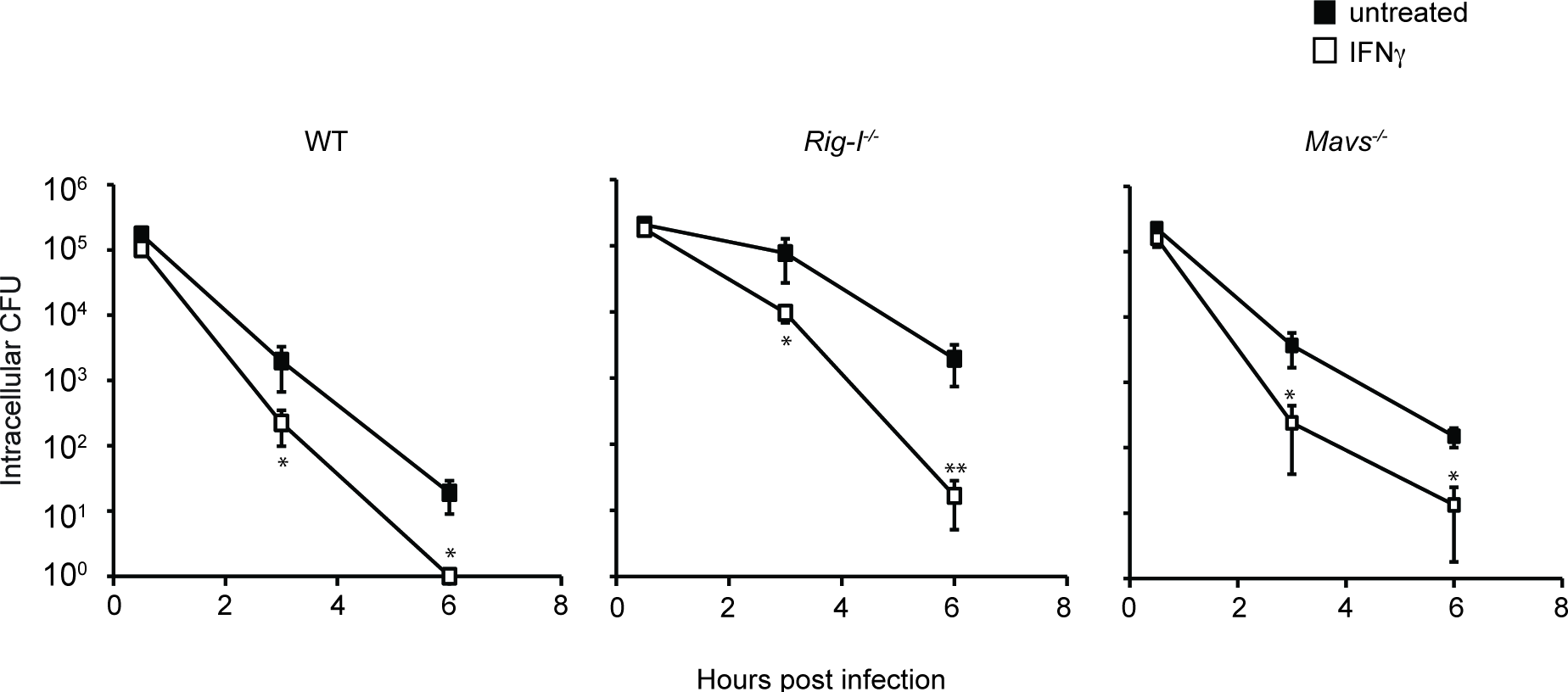

Supplement: Figure S2 — RIG-I and MAVS are dispensable for IFNγ-dependent restriction of S. flexneri in primary BMMs. Quantification of S. flexneri CFU in WT, Rig−I −, and Mavs−/− BMMs infected at an MOI of 1∶1 for indicated amounts of time. All data shown are means and standard deviations. Where appropriate, significant statistical differences are indicated as follows: ns, not significant; *, p<0.05; **, p<0.005 (Student's t test). Unless indicated otherwise, noted statistical differences are between unstimulated and IFNγ-stimulated cells for each condition. (TIF) [file ppat.1002809.s002.tif]
